# New findings of *Dunyu* (Eugaleaspiformes, Galeaspida) from the Xiaoxi Formation in South China and their biostratigraphic significance

**DOI:** 10.7717/peerj.18760

**Published:** 2024-12-24

**Authors:** Qiang Li, Xianren Shan, Zhikun Gai, Yang Chen, Lijian Peng, Jiaqi Zheng, Xianghong Lin, Wenjin Zhao, Min Zhu

**Affiliations:** 1 Research Center of Natural History and Culture, Qujing Normal University, Qujing, China; 2 Chongqing Institute of Geology and Mineral Resources, Chongqing, China; 3 Key Laboratory of Vertebrate Evolution and Human Origins, Institute of Vertebrate Paleontology and Paleoanthropology, Chinese Academy of Sciences, Beijing, China; 4 Key Laboratory of Yunnan Provincial Department of Education, Faculty of Biological Resource and Food Engineering, Qujing Normal University, Qujing, China; 5 University of Chinese Academy of Sciences, Beijing, China; 6 Yunnan Key Laboratory for Palaeobiology, Institute of Palaeontology, Yunnan University, Kunming, China

**Keywords:** Galeaspida, *Dunyu*, Xiaoxi formation, Biostratigraphy, Yangtze platform, South China

## Abstract

New discoveries of the late Silurian fossil fish *Dunyu* (Eugaleaspidae, Eugaleaspiformes, Galeaspida), *Dunyu tianlu* sp. nov. and *Dunyu* sp., are described from the Xiaoxi Formation in Xiushan of Chongqing and Xiushui of Jiangxi, China respectively. *D. tianlu* sp. nov. can be distinguished from *D. longiforus* and *D. xiushanensis* in its nearly equal preorbital and postorbital regions of the headshield. As the currently only known genus of Eugaleaspiformes during the late Silurian, *Dunyu* not only displays a large morphological difference with galeaspids from both the early Silurian and Early Devonian but also occupies a phylogenetic position that is far from the root of Eugaleaspiformes, which indicates that the lineages nested between Yongdongaspidae and Eugaleaspidae should have diversified before the early Ludlow, even during the Telychian. Discovery of new specimens of *Dunyu* provides direct evidence on the genus level for the correlation of the late Ludlow strata between the margin and interior of the Yangtze Platform, further supporting that the central part of the Yangtze Platform suffered from widespread transgression in the late Silurian.

## INTRODUCTION

The Siluro-Devonian Galeaspida is an endemic clade of jawless stem-gnathostomes, occurring exclusively in South China, North China, and Tarim blocks (Janvier, 1996; Zhu & Gai, 2006; Janvier et al., 2009; Sansom, 2009a; Gai et al., 2018). Fossil evidence shows that Galeaspida underwent early evolutionary radiation in South China and Tarim blocks during the Telychian (Llandovery, Silurian), diversifying into three deeply rooted groups (Dayongaspidae, Hanyangaspidae, and Xiushuiaspidae), along with Eugaleaspiformes (*e.g.*, Shuyuidae, Sinogaleaspidae, and Yongdongaspidae), as well as Polybranchiaspiformes (*e.g.*, Gumuaspidae) (Gai et al., 2018; Shan et al., 2020; Chen et al., 2022; Shan et al., 2022a; Shan et al., 2022b; Shan et al., 2023; Zhang et al., 2024). As a result of the Kwangsian Orogeny, the Yangtze Platform of the South China Block was uplifted by the end of the Telychian (Rong, Johnson & Yang, 1984; Rong et al., 1990; Rong et al., 2019), leading to a sedimentary gap ranging from the middle-late Telychian to the late Ludlow (approximately 10 million years). Consequently, the fossil record of galeaspids during this interval is absent. By the late Ludlow of Silurian, several areas of the Yangtze Platform had developed into residual basins, where the fossil record of galeaspids reoccurred alongside other early vertebrate fossils, *i.e.,* placoderms and osteichthyans of the Xiaoxiang vertebrate fauna (Zhu & Zhao, 2009; Zhu et al., 2009; Wang et al., 2010; Zhu et al., 2013; Zhao & Zhu, 2014; Zhao & Zhu, 2015; Zhu et al., 2016).

*Dunyu*, established based on the type species *Dunyu longiforus* from the Kuanti Formation in Qujing of Yunnan, China (Zhu et al., 2012), is the only known galeaspid fossil record from the Ludlow of Silurian. Therefore, the genus is of great significance in understanding the morphology and recovery of galeaspids during the late Silurian. Liu (1983) described *Eugaleaspis xiushanensis* from the late Ludlow Xiaoxi Formation (upper part of the Huixingshao or Xiaoxiyu Formation in Pan (1986)) in Xiushan of Chongqing, and this species used to represent the earliest occurrence of the genus *Eugaleaspis* (Liu, 1965; Liu, 1975). However, *E. xiushanensis* was later referred to as *Dunyu xiushanensis* based on the presence of the posteriorly extending cornual process, the headshield with its breadth/length ratio smaller than 1.1, and the median dorsal opening that is more posteriorly extended, a suit of characters that are absent in the Devonian *Eugaleaspis* (Zhu et al., 2012). Here we described a new species of *Dunyu, Dunyu tianlu* sp. nov., and *Dunyu* sp., from the Xiaoxi Formation in Xiushan of Chongqing and Xiushui of Jiangxi, China, respectively. The new discoveries not only enrich the diversity of galeaspids during the Ludlow but also provide additional evidence for the stratigraphic correlation between the Ludlow Red Beds (LDRBs) in South China.

## Material and Methods

### Material

The specimens of *Dunyu* in this study are permanently housed and accessible for examination in the collections of the Institute of Vertebrate Paleontology and Paleoanthropology, Chinese Academy of Sciences (IVPP). All fossil specimens were prepared mechanically using a vibro tool with a tungsten-carbide bit or a needle. They were measured with a digital vernier calliper, studied under optical zoom, and photographed with a Canon EOS 5D Mark III camera coupled with a Canon macro photolens (MP-E 65 mm 1:2.8 1–5 ×).

The specimens of *Dunyu tianlu* sp. nov. include a nearly complete headshield (IVPP V33246) and an incomplete headshield (IVPP V33247) that were collected from the Xiaoxi Formation in Tianlu scenic area of Xiushan County, Chongqing Municipality, China. The specimen of *Dunyu* sp. is an incomplete headshield (IVPP V30976) that was collected from the Xiaoxi Formation in Sidu Town of Xiushui County, Jiangxi Province, China. The Xiaoxi Formation, known as the Ludlow Red Beds, is mainly distributed in the interior of the Yangtze Platform of South China including central Guizhou, southeastern Chongqing, northwestern Hunan, southeastern Hubei, northwestern Jiangxi, and southwestern Anhui (Wang et al., 2010; Wang et al., 2011; Wang et al., 2017a; Wang et al., 2017b; Wang et al., 2018a; Wang et al., 2018b; Wang et al., 2018c). At both fossil localities of *Dunyu*, the Xiaoxi Formation shows disconformity in contact relationships with the underlying and overlying strata. The geological age of the Xiaoxi Formation is thought to be of late Ludlow, possibly extending to Pridoli, evidenced by nematophyte plants and micro-plant fossils (Wang et al., 2011; Wang et al., 2018a).

In the Xiushan area of Chongqing, the fish-bearing Xiaoxi Formation is dominated by grey-yellow and green-yellow sandstone, siltstone, silty mudstone, and mudstone (Li et al., 2021). The material of *Dunyu tianlu* sp. nov. was discovered from dark greenish-yellow silty mudstone near the top of the Xiaoxi Formation, approximately 9.8 m away from the bottom of the overlying Yuntaiguan Formation. In addition to plant debris, chitinozoans, and trace fossils, the associated fossils with *Dunyu tianlu* sp. nov. include the placoderm *Bianchengichthys micros* (Li et al., 2021). The Xiaoxi Formation disconformably overlies the Telychian Huixingshao Formation, in which the Chongqing Lagerstätte was found (Chen et al., 2022; Gai et al., 2022; Zhu et al., 2022).

In the Xiushui area of Jiangxi, the *Dunyu*-bearing Xiaoxi Formation mainly consists of yellow-green and grey-green fine-grained quartz sandstone interbedded with siltstone. The material of *Dunyu* sp. was collected from dark greenish-yellow silty mudstone near the top of the Xiaoxi Formation. This section is located 5 kilometers southwest of the Silurian Xikeng section. These two sections are located on the two wings of the same syncline, respectively. At the Xikeng section, the Xiaoxi Formation overlies the middle-late Telychian Xikeng Formation in which abundant galeaspids including *Sinogaleaspis*, *Rumporostralis*, and *Xiushuiaspis* are yielded (Pan & Wang, 1980; Pan & Wang, 1983; Gai et al., 2020; Shan et al., 2020). *Dunyu* sp. represents the first fossil fish discoveried in the late Ludlow Xiaoxi Formation in Jiangxi, China.

The electronic version of this article in Portable Document Format (PDF) will represent a published work according to the International Commission on Zoological Nomenclature (ICZN), and hence the new names contained in the electronic version are effectively published under that Code from the electronic edition alone. This published work and the nomenclatural acts it contains have been registered in ZooBank, the online registration system for the ICZN. The ZooBank LSIDs (Life Science Identifiers) can be resolved and the associated information viewed through any standard web browser by appending the LSID to the prefix http://zoobank.org/. The LSID for this publication is: urn:lsid:zoobank.org:pub: d7F540F5-C1A8-4C81-8599-A9004445FE3F. The LSID for *Dunyu tianlu* sp. nov. is: urn:lsid:zoobank.org:act:B5BE4DC7-0AE7-40F9-9FEB-DC2E3B5A29DB. The online version of this work is archived and available from the following digital repositories: PeerJ, PubMed Central and CLOCKSS.

### Phylogenetic analysis

To determine the phylogenetic position of *Dunyu tianlu* sp. nov. within Galeaspida, an extended phylogenetic analysis based on the updated dataset of Sun et al. (2022) and Liu et al. (2023) was conducted. Two new taxa, *Dunyu tianlu* sp. nov. and *D. xiushanensis*, were added to the data matrix (Supplemental Information). The phylogenetic character data entry and formatting were performed in Mesquite (version 3.61) (Maddison & Maddison, 2015). An early plesiomorphic osteostracan *Ateleaspis* was selected as the outgroup for the phylogenetic analysis (Sansom, 2009b). All characters were treated as unordered and weighted equally. The dataset was subjected to the maximum parsimony analysis in the TNT software package (Goloboff & Catalano, 2016). The analysis was conducted using a traditional search strategy, with the following settings: 10,000 maximum trees in memory and 1,000 replications.

## Results

### Systematic paleontology

**Table:** 

Subclass Galeaspida Tarlo, 1967
Order Eugaleaspiformes (Liu, 1965) Liu, 1980
Family Eugaleaspidae (Liu, 1965) Liu, 1980

**Differential diagnosis** **(emended).** Eugaleaspidae differs from all known galeaspids in its slit-like median dorsal opening that extends posteriorly nearly to or beyond the posterior margin of the orbital opening. It differs from other families of Eugaleaspiformes in the absence of the inner cornual process.

**Type genus.** *Eugaleaspis* (Liu, 1965) Liu, 1980

**Referred genera.** *Dunyu* Zhu et al., 2012; *Xitunaspis* Sun et al., 2022

Genus *Dunyu* Zhu et al., 2012

**Type species.** *Dunyu longiforus* Zhu et al., 2012

**Referred species.** *Dunyu xiushanensis* (Liu, 1983), *Dunyu tianlu* sp. nov.

**Differential diagnosis** **(emended).** *Dunyu* differs from other Eugaleaspiformes by the cornual process that extends posteriorly, a median dorsal opening extending posteriorly beyond orbital openings, and the strong size variation of polygonal flat-topping tubercles.

*Dunyu tianlu* sp. nov.

**Etymology.** After the Tianlu scenic zone, the fossil site.

**Holotype.** A nearly complete headshield, IVPP V33246a, and its external mould, IVPP V33246b.

**Referred specimens.** An incomplete headshield, IVPP V33247.

**Locality and horizon.** Tianlu scenic area, Xiushan County, Chongqing, China; Xiaoxi Formation, Ludfordian, late Ludlow, Silurian.

**Differential diagnosis.** *Dunyu tianlu* sp. nov. can be distinguished from other species of *Dunyu*, *D. longiforus* and *D. xiushanensis*, by the following characters: small-sized headshield with a maximum length of 43.2 mm and maximum width of 51.8 mm; length ratio between preorbital and postorbital regions of headshield approaching 1.0; third lateral transverse canal without a dichotomous end.

*Dunyu* sp.

**Material** An incomplete headshield, IVPP V30976.

**Locality and horizon.** Sidu Town, Xiushui County, Jiangxi, China; Xiaoxi Formation, Ludfordian, Ludlow, Silurian.

**Differential diagnosis.** *Dunyu* sp. differs from other species of *Dunyu* by the longer preorbital region (length ratio between the preorbital and postorbital portions of headshield perhaps greater than 1.0) and the closely related orbital openings with a distance of 13.2 mm between them.

**Remarks.** Information about the sensory canal system and the cornual process is unknown because of the poorly-preserved specimen.

### Description

### *Dunyu tianlu* sp. nov.

**Figure 1:**
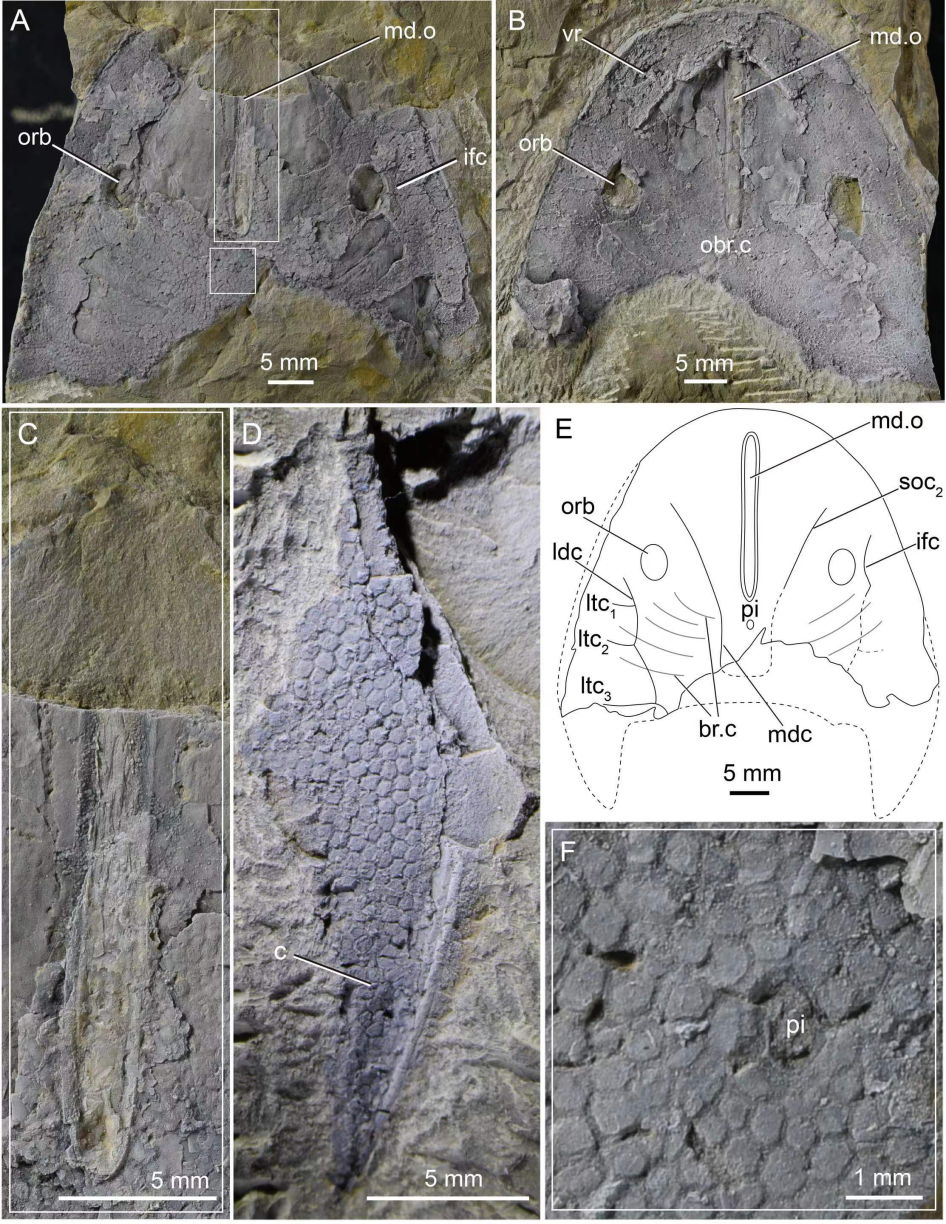
Photographs of *Dunyu tianlu* sp. nov. (A) A nearly complete internal mould of the headshield, holotype, IVPP V33246a. (B) A nearly complete external mould of the headshield, IVPP V33246b. (C) Box region of (A) showing close-up of dermal ring-like structure enclosing median dorsal opening. (D) A complete cornual process, IVPP V33247. (E) Interpretative drawings of (A). (F) Box region of (A) showing close-up of granular tubercles. Abbreviations: br.c, branchial chamber; c, cornual process; ifc, infraorbital canal; ldc, lateral dorsal canal; ltc, lateral transverse canal; mdc, median dorsal canal; md.o, median dorsal opening; obr.c, oralobranchial cavity; orb, orbital opening; pi, pineal opening; soc_2_, posterior supraorbital canal; vr, ventral rim.

**Figure 2:**
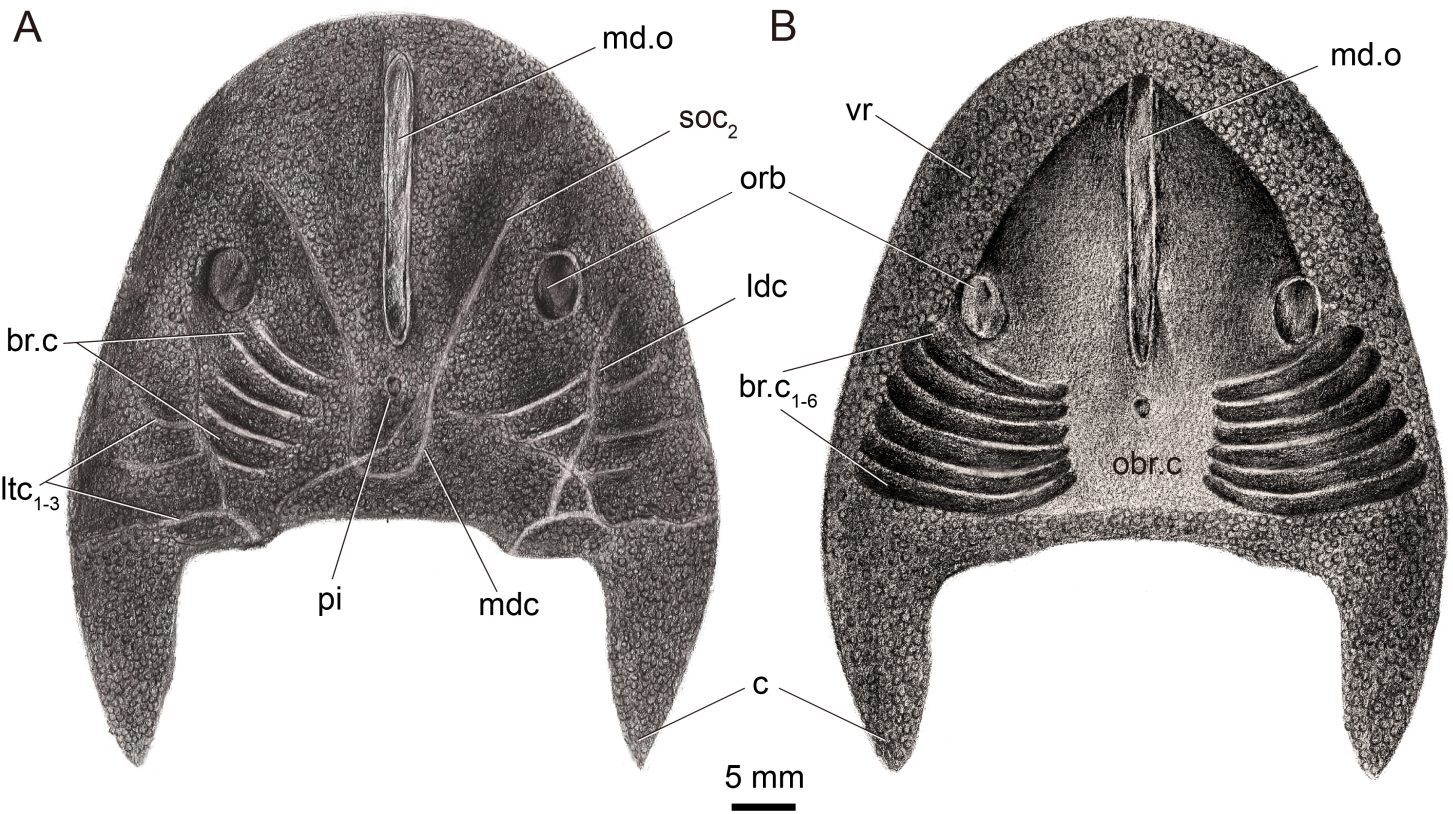
Restoration of *Dunyu tianlu* sp. nov. in dorsal (A) and ventral (B) views.

The holotype IVPP V33246 (Figs. 1A and 1B) that preserves a nearly complete dorsal headshield and partially ventral headshield, together with IVPP V33247 that preserves a cornual process (Fig. 1D), enables a reconstruction of a whole headshield morphology of *Dunyu tianlu* sp. nov. (Figs. 1E, 2A and 2B). The headshield has a medium size with a maximum width of 51.8 mm, a midline length of 43.2 mm, and an estimated maximum length of 56.2 mm (Table 1). The width-to-length ratio of the headshield is approximately 0.91, nearly equal to that of *Dunyu longiforus*, which is about 0.92. The rostral margin of the headshield is blunt arciform in outline without a rostral process or a rostral angle. The headshield attains its maximum width at nearly the base of the cornual process (c) where the lateral margin of the headshield is nearly parallel (Fig. 1A). The cornual process (Fig. 1D) is spine-shaped with a total length from its base to tip of approximately 13.0 mm. The inner cornual process is absent.

The median dorsal opening (md.o) (Figs. 1A, 1B, 1C and 2A) is longitudinal slit-like in outline with a length of 22.9 mm and a width ranging from 1.5 mm at its middle to 1.9 mm at its anterior and posterior ends (Table 1). The posterior end of the median dorsal opening extends posteriorly beyond the level of the posterior margin of orbital openings (orb). In the holotype, the dermal exoskeleton encircling the median dorsal opening is thickened, forming a ring-like structure (Figs. 1A and 1C). The pineal opening (pi) is situated 1.9 mm away from the posterior end of the median dorsal opening (Figs. 1A, 1E, 1F and 2A), and it is small with a diameter of 0.5 mm.

The orbital openings (orb) are dorsally positioned, oval in shape (Figs. 1A, 1B, 1E and 2A), and relatively large with a long axis at 5.2 mm and a short axis at 3.61 mm (Table 1). Each orbital opening is also encircled by a dermal ring-like structure. The distance between the medial margins of two orbital openings is approximately 26.5 mm. The length of the preorbital region, from the center of the orbital opening to the rostral margin, is 21.4 mm, while that of the postorbital region, from the center of the orbital opening to the posterior margin of the headshield (excluding the cornual process), is approximately 21.8 mm (Table 1). The length ratio between preorbital and postorbital regions is nearly 1.0.

The sensory canal system, which can be observed in the internal mould of the holotype (Figs. 1A and 1E), consists of infraorbital canal (ifc), lateral dorsal canals (ldc), lateral transverse canals (ltc), posterior supraorbital canal (soc_2_), median dorsal canal (mdc), and dorsal commissure (dcm). The distributing pattern of the sensory canal system of *Dunyu tianlu* sp. nov. is strikingly similar to that of the type species *D. longiforus*. Specifically, the infraorbital canal stars from the anterolateral side of the orbital opening, extending posteriorly and joining with the lateral dorsal canal at a bend (Figs. 1A and 1E). The lateral dorsal canal continues posteriorly to the posterior margin of the headshield. Three lateral transverse canals (ltc_1−3_) issue laterally from the lateral dorsal canals, and among them, the posteriormost one is much longer than the anterior two canals (Figs. 1A and 1E). The posterior supraorbital canal starts at the anterior side of the orbital opening, extends posteriorly towards the middle line of the headshield, and connects smoothly to the median dorsal canal at the level of the pineal opening (Figs. 1A and 1E). The paired median dorsal canals are nearly parallel and converge with the opposite one to form a U-shaped trajectory. One dorsal commissure, which is roughly in level with the second lateral transverse canal (ltc_2_), is present to connect the median dorsal canals and lateral dorsal canals (Figs. 1A and 1E). One dorsal commissure, which connects the lateral dorsal canals and median dorsal canals, should be present, but it cannot be clearly observed in the holotype (Figs. 1A and 1E).

**Table 1:** Measurements and comparisons of *Dunyu* (mm).

Items	*D. longiforus*	*D. xiushanensis*	*D. tianlu*	*D.* sp.
	IVPP V17681	V6793.1	V33246	V30976
Maximum length of the headshield	85.0	55.0	56.2	—
Maximum width of the headshield	78.0	58.0	51.1	49.2
Length of the headshield in midline	66.0	37.0	43.2	—
Long axis of orbital openings	10.0	5.0	5.2	4.2
Distance between orbital openings	—	20.0	21.1	13.1
Length of preorbital region in midline	28.0	16.5	21.4	29.8
Length of postorbital region in midline	38.0	20.5	21.8	—
Long axis of median dorsal opening	30.5	15.0	22.9	23.2
Short axis of median dorsal opening	1.6∼2.5	2.0	1.5∼1.9	1.5∼2.2
Diameter of pineal opening	2.0	—	0.5	0.9

In the holotype, the dorsal dermal skeleton posterior to orbital openings was partially destroyed, which resulted in six pairs of branchial chambers (br.c) naturally exposed and lined by five successive arranged shallow grooves (Figs. 1A and 1E). The dorsal headshield curves ventrally to form a flat ventral rim (vr) which is partially preserved in the external mould of the holotype (Fig. 1B). The anterior portion of the ventral rim is relatively broad with a width of approximately 8.0 mm on each side. In the central area of IVPP V33246b (Fig. 1B), there is a subtriangular depression enclosed by the ventral rim, indicating the position of the oralobranchial cavity (obr.c). The oralobranchial cavity comprises an anterior oronasal cavity that opens ventrally by an oral fenestra and a posterior branchial cavity that opens ventrally by several branchial openings (Gai et al., 2011). It is noteworthy that the anterior margin of the oral fenestra, defined by the posterior margin of the anteriormost ventral rim, is in an acute angle (Figs. 1B and 2B), a condition similar to that of *Dunyu longiforus* and *Falxcornus liui* (Meng & Gai, 2021) but distinct from that of basal galeaspids such as *Hanyangaspis guodingshanensis* (P’an, Wang & Liu, 1975) and *Changxingaspis gui* (Wang, 1991) in which the anterior margin of the oral fenestra is gently arched.

The ornamentation of the headshield consists of closely set, irregular, and polygonal tubercles, a condition similar to that of *Dunyu longiforus*. The tubercles show various sizes in different regions of the headshield. Specifically, the tubercles in the central area of the headshield are large (Figs. 1A and 1F), with a length of 0.9 mm, whereas those around the lateral margin of the headshield are relatively smaller, with a length of 0.6 mm.

**Figure 3:**
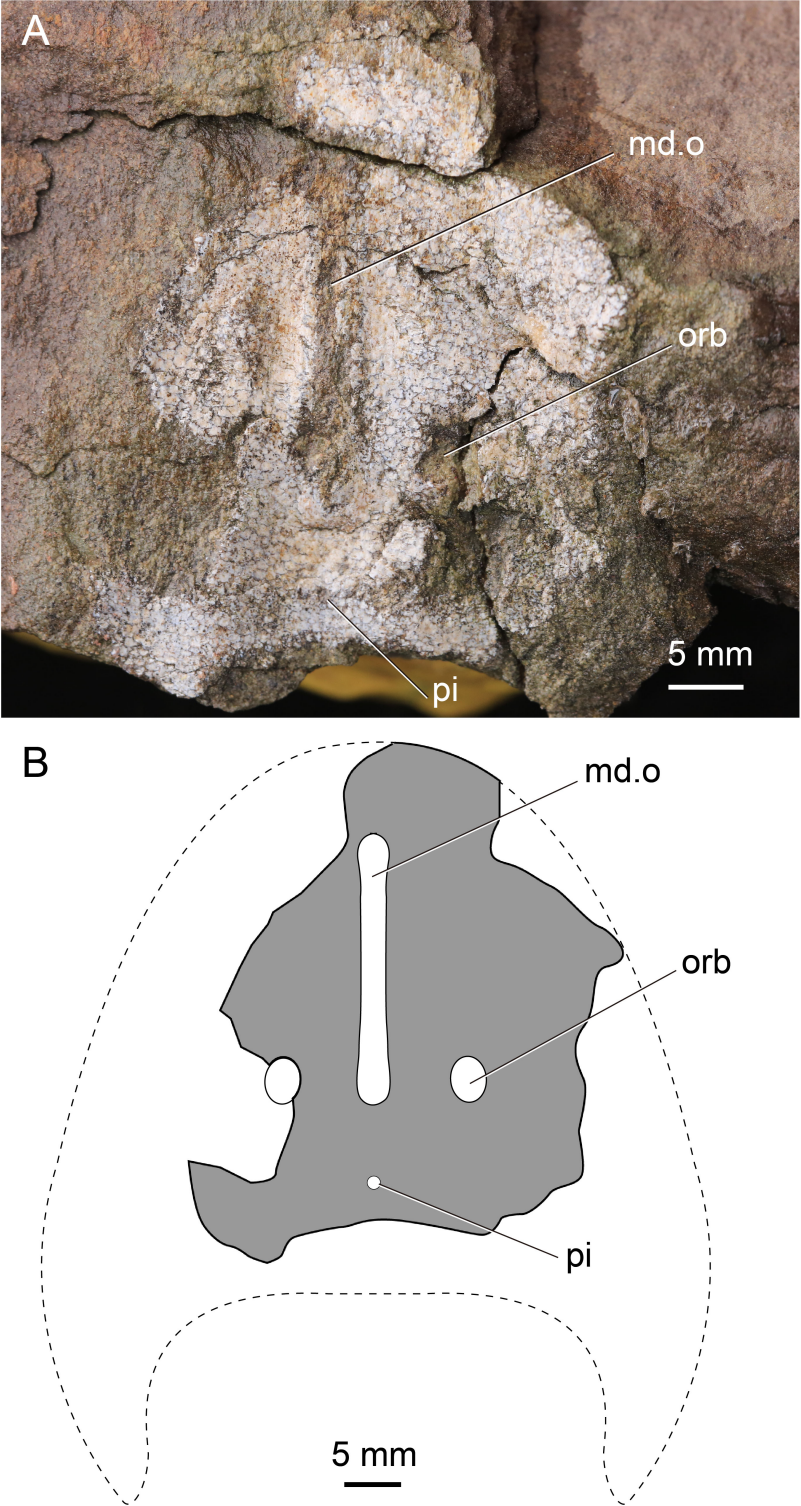
Photographs of *Dunyu* sp. (A) An incomplete external mould of the headshield, holotype, IVPP V30976. (B) interpretative drawings of (A).

### *Dunyu* sp.

The material of *Dunyu* sp. only includes an incomplete headshield with a preserved length of 46.3 mm and an estimated maximum width of 49.2 mm, suggesting that the whole length of the headshield is probably much greater than its width. The headshield width of *Dunyu* sp. approaches that of *D. tianlu* (51.1 mm) but is much smaller than that of *D. xiushanensis* (58.0 mm) and *D. longiforus* (78.0 mm). The median dorsal opening (Figs. 3A and 3B) is longitudinal slit-like in outline with a length of 23.2 mm and a width ranging from 1.5 mm to 2.2 mm (Table 1). The length-to-width ratio of the median dorsal opening is greater than 10.5. As in other species of *Dunyu*, the posterior end of the median dorsal opening extends posteriorly beyond the level of the posterior margin of orbital openings. The pineal opening (Figs. 3A and 3B) is far from the posterior end of the median dorsal opening with a distance of 7.0 mm between them. It is round in outline with a diameter of approximately 0.9 mm. The orbital openings (Figs. 3A and 3B) are dorsally positioned and relatively close to the midline of the headshield with a distance of 13.2 between them. The long axis of the orbital opening is nearly 4.2 mm (Table 1). The length of the preorbital region is 29.8 mm, approaching the length of *D. longiforus* (28.0 mm) but being much greater than the length of *D. tianlu* (21.4 mm) and *D. xiushanensis* (16.5 mm). The ornamentation of the headshield consists of closely set, polygonal, and large tubercles with the maximum length of a single tubercle exceeding 1.0 mm.

### Comparison

*Dunyu tianlu* sp. nov. can be assigned to the genus *Dunyu* because it exhibits a suit of diagnostic characters of the genus, including the median dorsal opening extending posteriorly beyond orbital openings, the posteriorly extending cornual process, and no inner cornual process. *D. tianlu* sp. nov. is more similar to *D. longiforus* than *D. xiushanensis* in the width-to-length ratio of the headshield, which is approximately 0.9, and in the length-to-width ratio of the median dorsal opening, which is nearly 12.0. Regarding individual size, *D. tianlu* sp. nov. approaches *D. xiushanensis* but is much smaller than *D. longiforus*. However, *D. tianlu* sp. nov. markedly differs from both *D. longiforus* and *D. xiushanensis* in the length ratio between the preorbital and postorbital regions of the headshield. The ratio is 0.99 in *D. tianlu* sp. nov., whereas it is 0.74 in *D. longiforus* and 0.80 in *D. xiushanensis*, which means that the orbital openings of *D. tianlu* sp. nov. are more posteriorly positioned than those of the latter two.

The specimen IVPP V30976 resembles *Dunyu*, *Xitunaspis*, and *Eugaleaspis* by the longitudinal slit-like median dorsal opening that extends to the posterior margin of orbital openings. However, *Xitunaspis* and *Eugaleaspis* are known exclusively from the Lochkovian to the Pragian of Lower Devonian in Yunnan and Guangxi, whereas IVPP V30976 is collected from the upper Ludlow Xiaoxi Formation in Jiangxi. By comparison, specimen IVPP V30976 is suggestive of the late Ludlow *D. tianlu* sp. nov. in headshield size, the length-to-width ratio of the median dorsal opening, and the ornamentation of the headshield. It only differs from *D. tianlu* sp. nov. in its longer preorbital region and more closely related orbital openings. Therefore, we propose to assign IVPP V30976 to *Dunyu*. Considering the lack of data on the sensory canal system and the cornual process, the erection of a new species is suspended for the specimen.

### Phylogenetic results

**Figure 4:**
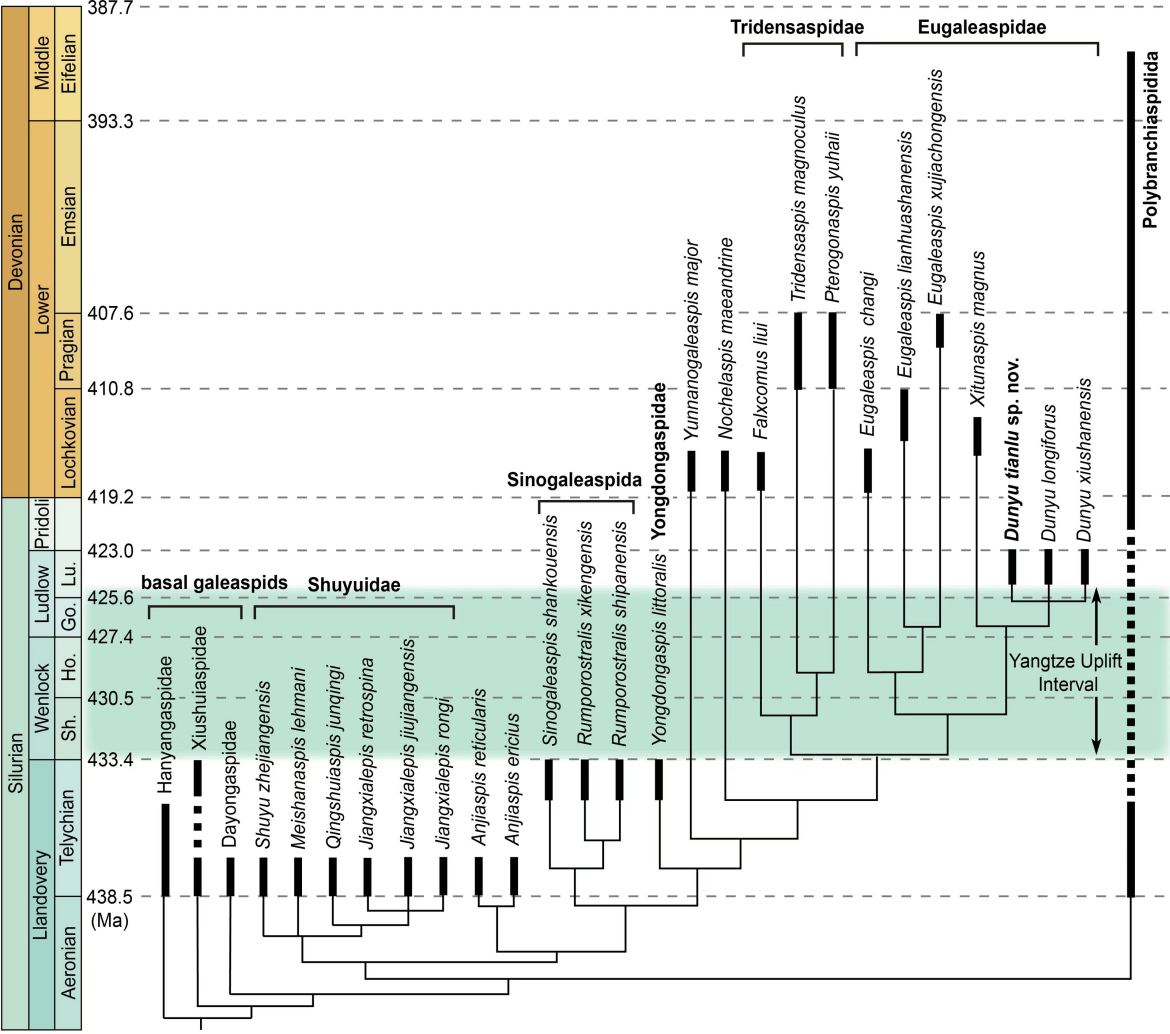
A simplified phylogenetic tree of galeaspid projected against stratigraphy. Eugaleaspiformes experienced two diversifications in Silurian and Devonian (Solid columns represent known time ranges, and thin lines represent ‘ghost lineages’) (data from (Sun et al., 2022). Abbreviations: Go., Gorstian; Ho., Homerian; Lu., Ludfordian; Sh., Sheinwoodian.

The maximum parsimony analysis produced five most parsimonious trees (Fig. 4) with a tree length = 216, consistency index (CI) = 0.389, and retention index (RI) = 0.781. A strict consensus tree shows that the monophyly of Eugaleaspidae consisting of *Eugaleaspis*, *Xitunaspis*, and *Dunyu* is supported by the loss of inner cornual processes (Fig. S1). Three species of *Dunyu* constitute a polytomy clade nested within Eugaleaspidae, supported by two synapomorphies including the posteriorly-projected cornual processes and the median dorsal opening extending posteriorly beyond the posterior margin of orbital openings.

## Discussion

### Taxonomic implications

*Dunyu tianlu* sp. nov. and *Dunyu* sp. enriched the morphological and taxonomic diversity of Eugaleaspidae and deepened the understanding of the distribution and diversity of galeaspids during the late Silurian. Eugaleaspidae was established by Liu (1965) based on the type genus *Eugaleaspis*. Zhu & Gai (2006) incorporated *Eugaleaspis*, *Yunnanogaleaspis* (Pan & Wang, 1980), *Pterogonaspis* (Zhu, 1992), *Tridensaspis* (Liu, 1986) and *Nochelaspis* (Zhu, 1992) into Eugaleaspidae based on the first cladistically-based classification of the Galeaspida. The following described *Dunyu* was also assigned to the Eugaleaspidae among which *Dunyu* was thought to be more closely related to *Eugaleaspis* than to other genera by the absence of inner cornual process (Zhu et al., 2012). However, the incorporation of *Yunnanogaleaspi* s, *Pterogonaspis*, *Tridensaspis*, and *Nochelaspis* into Eugaleaspidae, as proposed by Zhu & Gai (2006), will cause the diagnosis of the family to be greatly modified to occupy a larger morphospace (*e.g.*, bearing rostral process or not; inner cornual process absent or not; cornual process projecting laterally, posterolaterally, or posteriorly). Therefore, Shan et al. (2020) proposed to assign *Pterogonaspis* and *Tridensaspis* to the Tridensaspidae and remove *Yunnanogaleaspis* and *Nochelaspis* from the Eugaleaspidae to maintain the diagnostic stability of the Eugaleaspidae erected based on *Eugaleaspis* (Fig. 4). Among Eugaleaspiformes, the clade Eugaleaspidae was resolved as the highest branch by the synapomorphy of the loss of inner cornual processes, bearing a closer relationship to Tridensaspidae than *Yunnanogaleaspis* and *Nochelaspis*. The phylogenetic stability of Eugaleaspidae was also corroborated by the finding of the middle Lochkovian *Xitunaspis* which falls into the clade Eugaleaspidae and is sister to *Dunyu* (Sun et al., 2022). *Dunyu tianlu* sp. nov. described herein displays nearly equal preorbital and postorbital regions, which is unique among Eugaleaspidae, thus increasing the taxonomic and morphological diversity of the clade during the Ludlow of late Silurian.

Fossil records show that the Eugaleaspiformes diverged from the basal galeaspids as early as the Telychian (Llandovery, Silurian) during which they reached the highest taxonomic diversity with the occurrence of Shuyuidae, Sinogaleaspida, Yongdongaspidae, and *Anjiaspis* (Chen et al., 2022; Shan et al., 2022a; Shan et al., 2022b). After a major decline caused by the Yangtze Uplift, the diversity of Eugaleaspiformes gained a second peak during the Early Devonian (Fig. 4). As a stratigraphically intermediate member, however, *Dunyu* fails to fill the morphological gap of Eugaleaspiformes between the early Silurian and Early Devonian. By contrast, it exhibits a large number of specialized features, such as the absence of the inner cornual process and the posteriorly extending cornual process. Recent phylogenetic results resolve *Dunyu* as a sister group to *Xitunaspis* (Sun et al., 2022), a phylogenetic position that is far from the root of Eugaleaspiformes, indicating the lineages positioned between Yongdongaspidae and Eugaleaspidae should have diversified before the early Ludlow, even during the Telychian (Fig. 4).

**Figure 5:**
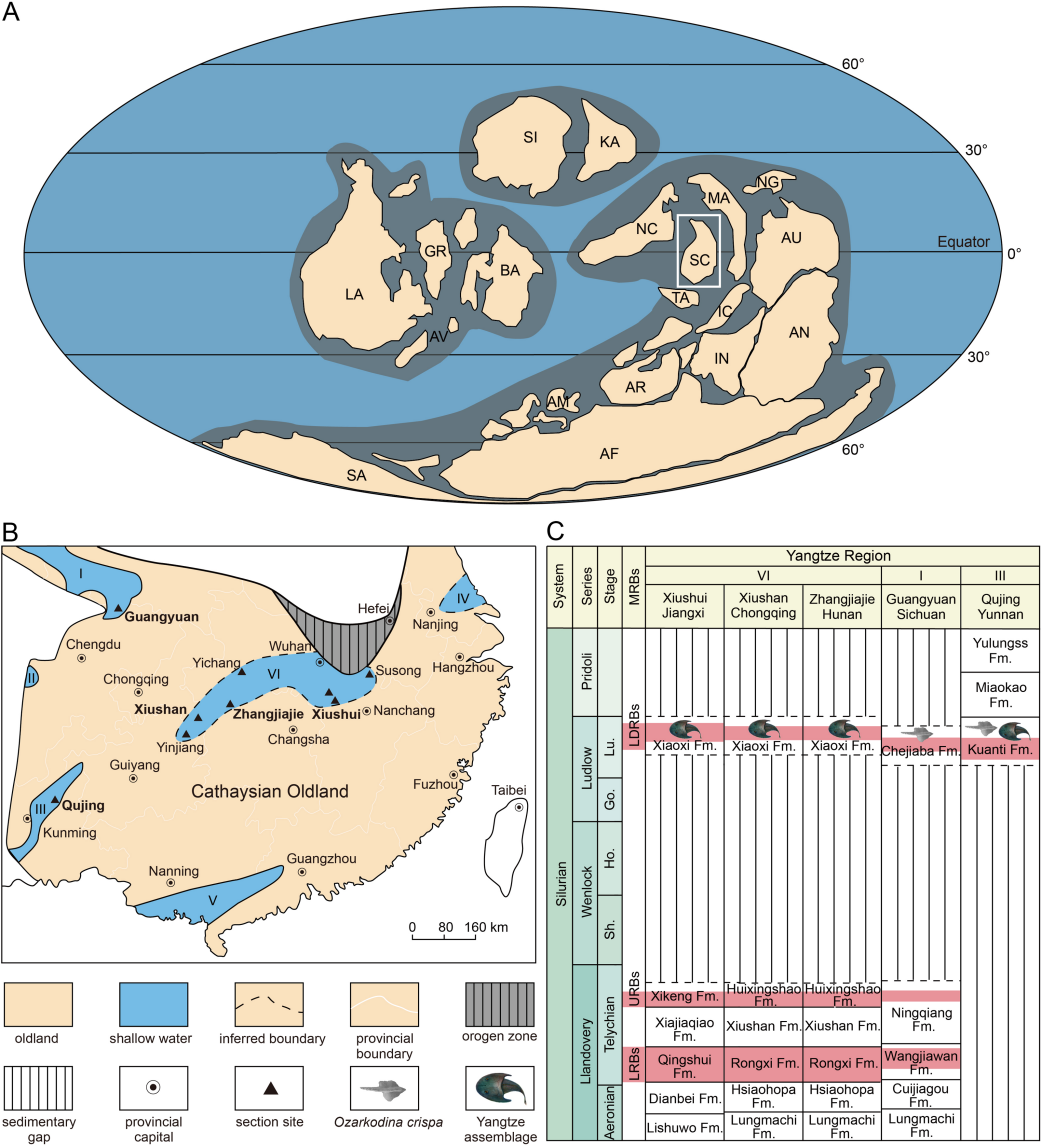
Reconstructing of global plates distribution during Silurian showing the position of South China (A), paleogeographic map of South China during late Silurian (B), and the stratigraphical positions and correlations of the Ludlow Red Beds in Yangtze Region. (A) is modified from Liu et al. (2023). (B) is based on the data from Rong et al. (2019). (C) is modified from Shan et al. (2022b). Abbreviations: I, northern Sichuan; II, western Sichuan; III, eastern Yunnan, IV, northern Jiangsu; V, southern Guangxi and Guangdong; VI, interior of the Yangtze Platform; AF, Africa, AM, Armorica, AN, Antarctic, AR, Arabia; AU, Australia; AV, Avalonia, BA, Baltica, Fm., Formation; GR, Greenland, IC, Indochina, IN, India; KA, Kazakhstan; LA, Laurentia; LRBs, Lower Red Beds; LDRBs, Ludlow Red Beds; MA, Malaya; MRBs, marine red beds; NC, North China; NG, New Guinea; SA, South America, SC, South China; SI, Siberia; TA, Tarim; URBs, Upper Red Beds.

### Biostratigraphic significance

The Silurian shallow marine red beds are widely distributed in South China (Fig. 5A), and three sets of them, informally called the Lower Red Beds (LRBs), the Upper Red Beds (URBs), and the Ludlow Red Beds (LDRBs), have been recognized mainly in the following three horizons: the lower Telychian, the upper Telychian, and the upper Ludlow (Rong, Wang & Zhang, 2012; Rong et al., 2019). For a long time, the late Ludlow strata were considered to be distributed exclusively in the marginal area of the Yangtze Platform of South China Block (Rong et al., 2003), including western and northern Sichuan (Jin et al., 1989; Wan et al., 1991), eastern Yunnan (Ge et al., 1979; Wang, 2001), northern Jiangsu (Geng et al., 1997; Wang & Li, 2000; Wang & Li, 2001), and southern Guangxi and Guangdong (Fig. 5B). This paleogeographic pattern was caused by the “Yangtze Uplift” that resulted in the Yangtze Platform of South China Block as a whole being uplifted by the end of the Telychian (Llandovery, Silurian) (Rong, Johnson & Yang, 1984; Rong et al., 1990). In the past two decades, the late Ludlow shallow marine deposits (known as the Xiaoxi Formation) were successively discovered in the interior of the Yangtze Platform including Xiushan of Chongqing, Zhangjiajie of Hunan, Yinjiang of Guizhou, Yichang and Tongshan of Hubei, Xiushui of Jiangxi, and Susong of Anhui, indicating that the shallow seawater invaded into the central part of the Yangtze Platform during the late Ludlow after the “Yangtze Uplift” (Fig. 5B) (Wang et al., 2010; Wang et al., 2011; Wang et al., 2017a; Wang et al., 2017b; Wang et al., 2018a; Wang et al., 2018b; Wang et al., 2018c).

The age of the late Silurian rocks along the margin of the Yangtze Platform (Region I and III, Fig. 5B) can be determined by the conodonts like *Ozarkodina snajdri* and *O. crispa*, as well as the brachiopods like the *Retziella* fauna (Wan et al., 1991; Jin et al., 1992; Tang et al., 2010). For example, the Chejiaba Formation in northern Sichuan is mainly of late Ludlow age and could extend upward to lower Pridoli (Tang et al., 2010; Wang et al., 2017b). However, the common marine index fossils are nearly absent in the Ludlow deposits (Xiaoxi Formation) in the interior of the Yangtze Platform (Region VI, Fig. 5B) where the recognition and correlation of the Ludlow strata have been weakly supported by nematophytes, tubular trace fossils, as well as fossil fishes (Zhao & Zhu, 2010; Wang et al., 2017b).

In Qujing of East Yunnan, the Ludlow Red Beds (LDRBs) are found in the Kuanti Formation, which has yielded the Yangtze Vertebrate Assemblage represented by galeaspids *Dunyu longiforus*, placoderms *Entelognathus primordialis* and *Qilinyu rostrata* (Zhu et al., 2013; Zhu et al., 2016), and osteichthyans *Guiyu oneiros* and *Megamastax amblyodus* (Zhao & Zhu, 2010; Zhao & Zhu, 2014). This assemblage can be confidently assigned to the Ludfordian age (Ludlow, Silurian) because it is immediately beneath the first appearance of *Ozarkodina crispa* (Zhao & Zhu, 2014; Zhao & Zhu, 2015; Cai, Zhao & Zhu, 2020). Therefore, the Yangtze Vertebrate Assemblage can provide a palaeoichthyological standard for the correlations of the Ludlow Red Beds (LDRBs) between the margin and interior of the Yangtze Platform.

**Figure 6:**
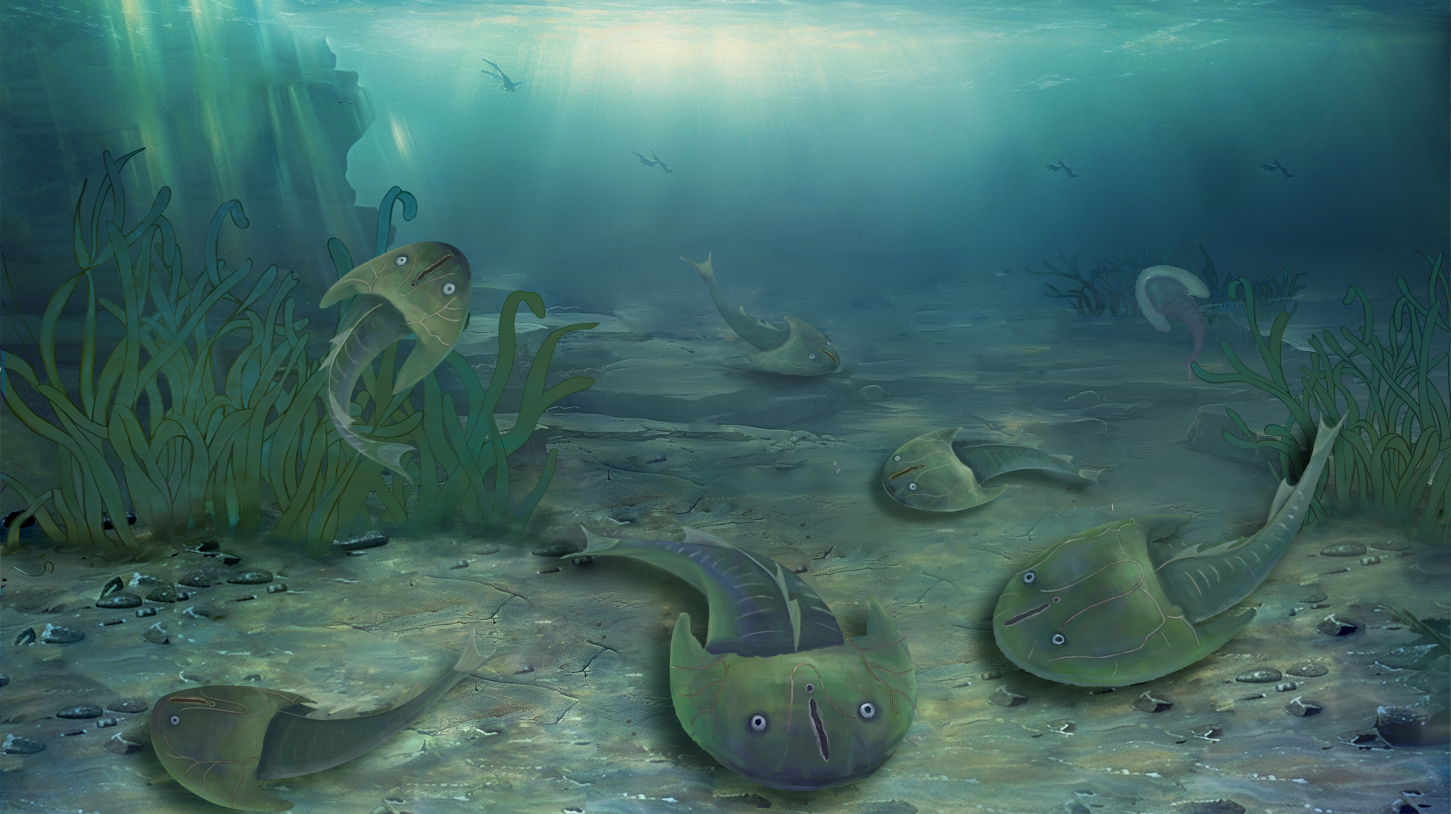
Life restoration of *Dunyu tianlu* sp. nov. Artwork credit: Jinjing Li.

Previously, mainly based on *Dunyu*, the correlation between the Kuanti Formation in Qujing of Yunnan and the Xiaoxi Formation in Xiushan of Chongqing was suggested (Zhao & Zhu, 2014; Zhao & Zhu, 2015). However, the horizon of the *Dunyu xiushanensis* in Xiushan of Chongqing was in reality not clear in Liu (1983), and it was inferred to be the upper member of the ‘Huixingshao Formation’ (corresponding to the Xiaoxi Formation) by Pan (1986). *Dunyu tianlu* sp. nov. described herein confirmed that the strata yielding *Dunyu* in Xiushan of Chongqing belong to the Xiaoxi Formation, thus providing new evidence for the reliable correlation between the Xiaoxi Formation and the Kuanti Formation (Fig. 5C). The funding of *Dunyu* sp. expanded the distribution of *Dunyu* to northwestern Jiangxi and provided further evidence for the correlation between the Xiaoxi Formation and the Kuanti Formation in eastern Yunnan. Therefore, the genus *Dunyu* is of great biostratigraphic significance in the recognition and correlation of the upper Ludlow in South China (Fig. 5C). The paleogeographic distribution of the late Ludlow *Dunyu* in the interior of the Yangtze Platform (Fig. 6) also corroborates that the central part of the Yangtze Platform suffered from widespread transgression in the late Silurian.

## CONCLUSIONS

The new materials of eugaleaspids from the upper Silurian of Chongqing, China, provide reliable diagnostic features for the erection of a new species, *Dunyu tianlu* sp. nov. *Dunyu* shows a large morphological difference to all other eugaleaspiform members and phylogenetically forms a sister to the Devonian *Xitunaspis*, which indicates that the lineages phylogenetically between Yongdongaspidae and Eugaleaspidae should have diversified before the early Ludlow, even during the Telychian. The occurrences of *Dunyu* from the Xiaoxi Formation in Chongqing and Jiangxi and the Kuanti Formation in Yunnan provide reliable evidence for the correlations of the Ludlow Red Beds (LDRBs) between the margin and interior of the Yangtze Platform of South China.

## Supplemental Information

10.7717/peerj.18760/supp-1 Supplemental Information 1 Strict consensus tree of 5 most parsimonious trees showing the phylogenetic position of *Dunyu tianlu* with Galeaspida Tree length = 216, consistency index (CI) = 0.389, retention index (RI) = 0.781. Numbers on branches denote bootstrap frequencies (below node) and Bremer support values (above node), bootstrap frequencies below 50 are not shown.

10.7717/peerj.18760/supp-2 Supplemental Information 2 Character description,

10.7717/peerj.18760/supp-3 Supplemental Information 3 Data matrix for the phylogenetic analysis of Galeaspida

## Additional Information and Declarations

### Data Availability

The following information was supplied regarding data availability: The two headshields of *Dunyu tianlu* sp. nov. (IVPP V33246 and V33247) and one headshield of *Dunyu* sp. (IVPP V30976), are permanently housed and accessible for examination in the collections of the Institute of Vertebrate Paleontology and Paleoanthropology (IVPP), Chinese Academy of Sciences.

### New Species Registration

The following information was supplied regarding the registration of a newly described species: Publication LSID: urn:lsid:zoobank.org:pub:D7F540F5-C1A8-4C81-8599-A9004445FE3F. Dunyu tianlu species LSID: urn:lsid:zoobank.org:act:B5BE4DC7-0AE7-40F9-9FEB-DC2E3B5A29DB.
