# Supplementary figures and images for: New findings of Dunyu (Eugaleaspiformes, Galeaspida) from the Xiaoxi Formation in South China and their biostratigraphic significance
Source: PeerJ. 2024 Dec 24;12:e18760. doi: 10.7717/peerj.18760 (PMC11674142; doi:10.7717/peerj.18760)

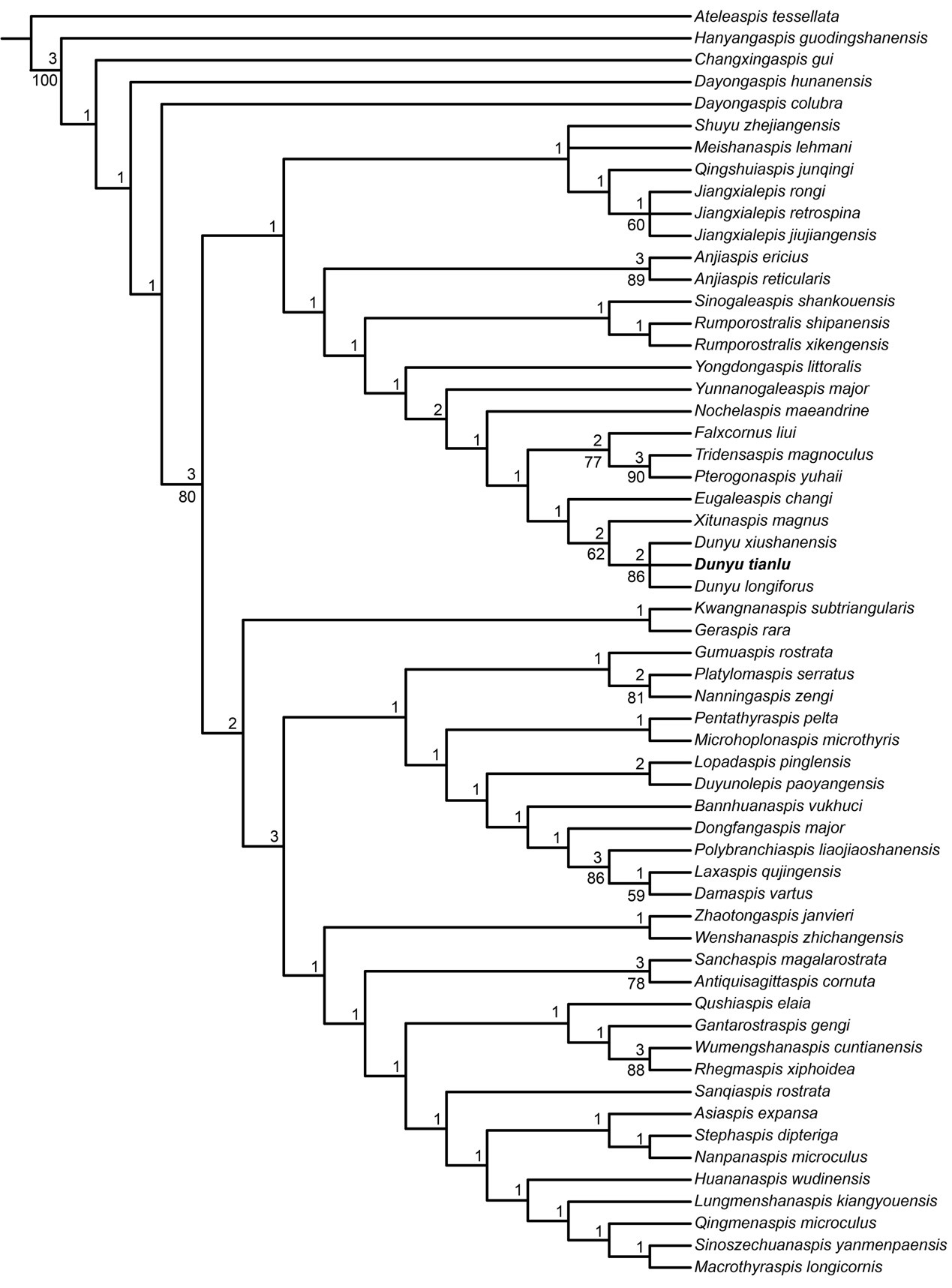

Supplement: Supplemental Information 1 — Tree length = 216, consistency index (CI) = 0.389, retention index (RI) = 0.781. Numbers on branches denote bootstrap frequencies (below node) and Bremer support values (above node), bootstrap frequencies below 50 are not shown. [file peerj-12-18760-s001.jpg]
