# Supplementary material for: New findings of Dunyu (Eugaleaspiformes, Galeaspida) from the Xiaoxi Formation in South China and their biostratigraphic significance
Source: PeerJ. 2024 Dec 24;12:e18760. doi: 10.7717/peerj.18760 (PMC11674142; doi:10.7717/peerj.18760)
Supplement: Supplemental Information 2 [file peerj-12-18760-s002.docx]

**Character description**

1. Median dorsal opening: (0) absent; (1) present.

[2] Festooned pattern of sensory canals on dorsal surface of headshield: (0) absent; (1) present.

[3] Aspidine tubercles: (0) absent; (1) present.

[4] Shape of headshield: (0) trapezoid-like; (1) triangular-like; (2) oval-like; (3) armet-like; (4) fork-like

[5] Margin of headshield: (0) serrated; (1) smooth.

[6] Shape of median dorsal opening: (0) transverse slit-like; (1) oval-like; (2) slender longitudinal oval（3）transverse crescent

1. Longitudinal oval dorsal opening: (0) not slit-like (length/width<6); (1) slit-like (length/width>6).
2. Heart-shaped median dorsal opening: (0) absent; (1) present.

[9] Position of median dorsal opening: (0) not subterminal; (1) subterminal.

[10] Anterior end of median dorsal opening: (0) subterminal; (1) some distance behind rostral margin of shield; (2) terminal.

[11] Posterior end of median dorsal opening: (0) in front of or level with anterior margin of orbital opening; (1) between anterior margin of orbital opening and posterior margin of orbital opening; (2) posteriorly beyond posterior margin of orbital opening.

[12] Size of orbital openings: (0) large; (1) small.

[13] Position of orbital openings: (0) dorsal position and close to mid-line of headshield; (1) dorsal position and not close to mid-line of headshield; (2) lateral position; (3) ventrolaterally placed orbital openings

[14] Cornual process: (0) absent; (1) present.

[15] Extending trajectory of cornual process: (0) projecting posterolaterally; (1) projecting laterally; (2) projecting backward.

[16] Portion of headshield behind cornual process: (0) short; (1) long.

[17] Position of cornual process base: (0) near posterior end of headshield; (1) away from posterior end of headshield.

[18] Spines on cornual process: (0) absent; (1) present.

[19] Inner cornual process: (0) present; (1) absent.

[20] Shape of inner 0cornual process: (0) broad leaf-shaped; (1) spine-shaped.

[21] Rostral margin of headshield: (0) without rostral angle; (1) with rostral angle.

[22] Rostral process: (0) absent; (1) present.

[23] Shape of rostral process: (0) broad; (1) slender.

[24] Spines or tubercles on margin of rostral process: (0) absent; (1) present.

[25] Fenestrae on dorsal surface of headshield: (0) absent; (1) present.

[26] Size of dorsal fenestra: (0) small; (1) large.

[27] Shape of dorsal fenestra: (0) slender oval-like; (1) broad bean-like.

[28] Position of dorsal fenestra: (0) lateral-dorsal position; (1) dorsal position.

[29] Position of dorsal fenestra relative to orbital opening: (0) orbital opening inside, fenestra outside; (1) orbital opening outside, fenestra inside.

[30] Median transverse canals (mtc): (0) two; (1) one; (2) more than two.

[31] Short branches running from posterior supraorbital canal (soc2): (0) absent; (1) present.

[32] Lateral transverse canal: (0) short; (1) long.

[33] Branching end of lateral transverse canal: (0) absent; (1) present.

[34] Lateral transverse canals leaving from infraorbital canal: (0) present; (1) absent.

[35] The lateral transverse canals leaving from infraorbital canal before ltc_d_: (0) present; (1) absent.

[36] Fourth lateral transverse canal (ltc4): (0) present; (1) absent.

[37] Lateral transverse canals behind ltc4: (0) present; (1) absent.

[38] Anterior supraorbital canal (soc1): (0) absent; (1) present.

[39] Posterior supraorbital canal (soc2): (0) present; (1) absent.

[40] Posterior supraorbital canals (soc2) meet with infraorbital canal (ifc): (0) no; (1) yes.

[41] Posterior supraorbital canals (soc2): (0) funnel-shaped; (1) parallel; (2) V-shaped.

[42] Branching end of posterior supraorbital canals (soc2): (0) absent; (1) present.

[43] Medial dorsal canal (mdc): (0) develeped; (1) degenerated; (2) absent.

[44] Medial dorsal canal and posterior supraorbital canal: (0) unjointed; (1) contact.

[45] U-shaped medial dorsal canal: (0) absent; (1) present.

[46] Portion of headshield behind dorsal commissure proportionally: (0) long; (1) short.

[47] Postbranchial wall: (0) short; (1) long.

[48] Elongated branchial region: (0) absent; (1) present.

[49] Number of branchial fossae: (0) 5~7 pairs; (1) 9~17 pairs; (2) more than 20 pairs.

[50] Maximum width of headshield placed: (0) posteriorly; (1) medially.

[51] Width/length in oval-like headshield: (0) < 1; (1) >1.

[52] Nearly parallel lateral margins of headshield: (0) absent; (1) present.

[53] Broad and large middle dorsal spine of headshield: (0) absent; (1) present.

[54] Broad ventral rim: (0) absent; (1) present.

[55] The unclosed rostral margin: (0) present; (1) absent.

[56] The ornamentation of the head-shield: (0) star-shaped tubercles; (1) tiny granular tubercles; (2) coarse granular tubercles；(3) polygon-shaped tubercles;(4) central big tubercles surronded by radially arranged tubercles.

[57] Pineal organ: (0) on front of or level with posterior margin of orbital opening; (1) behind posterior margin of orbital opening.

[58] Ratio between pre-pineal length and post-pineal length in mid-line of cephalic shield larger than 1.0: (0) no; (1) yes.

[59] The preorbital commissure：(0) absent; (1) presen

[60] Ventrally curved branchial fossae: (0) absent; (1) present

[61] Ventral rim of head-shield: (0) absent; (1) present

[62] Sickle-like complex of cornual and inner cornual processes (the angle of the cornual and inner cornual processes is equal to or more than 90 degrees): (0) absent; (1) present

[63] The inner cornual process exceeds the posterior edge of the cornual process in the galeaspids with triangular headshields: (0) absent; (1) present

[64] Middle dorsal spine of headshield: (0) absent; (1) present

[65] Central canal: (0) absent; (1) present

[66] Dermal bone of headshield: (0) no less than 1 mm in thickness; (1) less than 1 mm in thickness

[67] Vestige of median transverse canal: (0) absent; (1) present
